# CT and MR in peritoneal malignancies: pearls and pitfalls at preoperative examination

**DOI:** 10.1186/s13244-025-02060-z

**Published:** 2025-08-08

**Authors:** Michela Polici, Federica Palmeri, Erica Golia, Emanuela Parri, Guido Gentiloni Silveri, Francesco Puglisi, Domenico De Santis, Marta Zerunian, Francesco Pucciarelli, Tiziano Polidori, Maria Ciolina, Paolo Sammartino, Andrea Laghi, Damiano Caruso

**Affiliations:** 1 Department of Medical-Surgical Sciences and Translational Medicine, Sapienza University of Rome - Sant’Andrea University Hospital, Rome, Italy; 2 PhD School in Translational Medicine and Oncology, Department of Medical-Surgical Sciences and Translational Medicine, Faculty of Medicine and Psychology, Sapienza University of Rome, Rome, Italy; 3 Cytoreductive Surgery and HIPEC Unit, Department of Surgery “Pietro Valdoni”, Sapienza University of Rome, Rome, Italy; 4 Department of Biomedical Sciences, Humanitas University, Milan, Italy; 5 Department of Diagnostic Imaging, IRCCS Humanitas Research Hospital, Rozzano, Italy

**Keywords:** Peritoneum, Radiology, General surgery, CT, MRI

## Abstract

**Abstract:**

Peritoneal malignancies (PM) are defined as the spread of malignant epithelial cells in the peritoneal cavity. Until the recent past, the prognosis was considered extremely poor, and the treatment options had only palliative intent. Currently, new locoregional treatments have radically changed the outcome. CT is pivotal in PM diagnosis, staging, surgical planning, and determining therapeutic decisions. MRI should be evaluated in a preoperative setting for the evaluation of mesentery, serosal, and in any cases of contraindication of CT with contrast medium, while in the restaging clinical setting, it does not have a defined role. In the preoperative clinical setting, imaging could provide the surgeon with specific information concerning disease burden by showing the invasion of vital anatomic structures, and it is therefore essential to describe the feasibility of the surgery. However, recognizing the imaging findings of peritoneal deposits depends mainly on the histology of the primary tumor and the peritoneal spaces, thus rendering knowledge of peritoneal anatomy essential. In addition, some benign pathologies show similar imaging features that overlap with PM, making differential diagnosis difficult. It is still unclear which of the two methods, CT and MRI, is superior in terms of performance, and literature data are often controversial. Thus, the purpose of this review is to provide some practical tips for CT and MRI protocols and imaging findings essential to detect and characterize peritoneal deposits in each anatomical space, and to provide an overview of the main differential diagnosis with other peritoneal conditions.

**Critical relevance statement:**

Peritoneal malignancies should be understood as a heterogeneous pattern of diseases, with variable prognosis and treatment options. CT remains the main imaging method; MRI finds application for involvement of the serosa and mesentery and when contrast-enhanced CT is not feasible.

**Key Points:**

- CT is the first imaging option to assess peritoneal malignancies and plan surgery; however, they have several limitations, especially in critical regions.
- MRI could be seen as a supporting imaging approach in a preoperative setting to study serosal, mesentery, and in case of contraindication of CT with contrast medium.
- Multidisciplinary approach should always be considered in the assessment of peritoneal malignancies due to their heterogeneity.

**Graphical Abstract:**

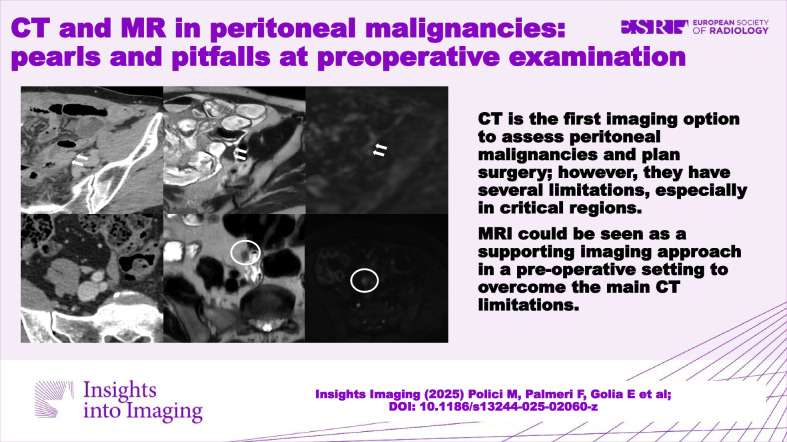

## Introduction

Peritoneal involvement can result from primary or metastatic disease [1]. Rarely is the peritoneum affected by primary tumors originating from mesothelial cells, which commonly have a better prognosis than peritoneal carcinomatosis (PC). Secondary involvement of the peritoneum is the most common peritoneal involvement, known as PC, in which the primary tumor arises from various cancers, mainly gastrointestinal or gynecological [2]. PC is associated with an inferior prognosis, with an average survival from 6 months to 5 years, due to the aggressiveness of the primary cancer [3, 4]. In the last few years, the PC prognosis has slightly improved due to the new proposals in which the combination of systemic chemotherapy, hyperthermic intraperitoneal chemotherapy, and cytoreductive surgery takes the lead [2]. From an imaging point of view, CT remains the first option to study the extension of malignant peritoneal malignancies (PM) due to its availability, cost-effectiveness, and spatial resolution, but it is limited in contrast resolution [5]. MRI could be seen as a problem solver to improve imaging performance in terms of sensitivity and specificity only in dedicated anatomical regions (e.g., small bowel or pelvis) [6]. New international guidelines were published by the major international societies [7, 8], indicating CT as the mandatory imaging approach, even considering that MRI and PET/CT might be seen as two complementary methods. However, while MRI has a definite role in staging, especially in the study of dedicated anatomic regions or as an alternative to contrast-enhanced CT, PET has a role only in cases of doubtful metastatic lymph nodes, high suspicion of persistent disease undetectable at CT, and in peritoneal pseudomyxoma [7, 8]. Thus, this review aims to provide an overview of the main cornerstones in peritoneal malignancies, focusing on anatomy, histology, CT and MRI protocol, imaging findings, pitfalls, and future perspectives.

## Anatomy and histology

**Fig. 1:**
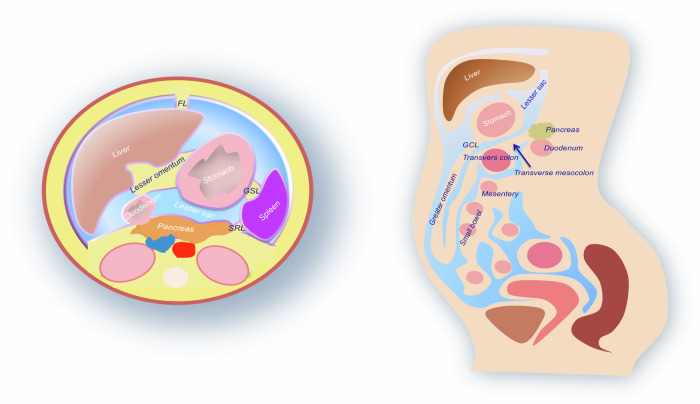
Anatomic scheme representation of peritoneal folds, ligaments and abdominal spaces. FL, falciform ligament; GSL, gastro-splenic ligament; SRL, splenic-renal ligament; GCL, gastro-colic ligament

The peritoneum is a serous membrane that covers most of the abdominal organs. It has two main layers: the parietal peritoneum and the visceral peritoneum. Between these layers, there is the peritoneal cavity that contains the serous fluid. The transverse colon divides the abdominal cavity into supra and inframesocolic space. The former knows a right and left supramesocolic space, separated by a falciform ligament. Gastrohepatic and hepatoduodenal ligaments form the lesser omentum. The oblique small bowel mesentery divides the inframesocolic space. The right paracolic gutter is continuous perihepatic space, and the left paracolic gutter is divided from the left subphrenic space by the phrenicocolic ligament. Supra and inframesocolic spaces communicate thanks to the right paracolic gutter. Regarding the pelvis, peritoneal reflections create the midline recto-vesical pouch in males, the recto-uterine pouch (pouch of Douglas) in females, and the paravesical fossae (Fig. 1) [9].

Peritoneal implants usually occur as secondary lesions, mainly from gastrointestinal or ovarian cancer and rarely from breast or lung cancers, each one with different histological findings that translate into several imaging characteristics. Mostly, peritoneal implants are solid with reduced enhancement in almost all histology, except for mucinous histology, which could manifest as cystic or calcified lesions, and neuroendocrine tumors, which could appear as hyperattenuating lesions in the arterial and portal venous phase [10]. Moreover, a different entity, originating from the perforated epithelial neoplasm of the appendix, known as pseudomyxoma peritonei (PMP), is usually characterized by cystic lesions with mucinous patterns with a variable aggressive behavior [11].

Among the primary malignancies of the peritoneum, mesothelioma is the most common, followed by primary peritoneal papillary serous carcinoma or primary peritoneal carcinoma [12, 13]. Peritoneal mesothelioma is known in two variants, benign and malignant, originating from the pleura’s mesothelial cells and, most rarely, from the peritoneum, pericardium, tunica vaginalis testis, and ovarium [12]. There are three most prevalent presentations of malignant peritoneal mesothelioma: dry type, wet type, and mixed type, according to the presence of ascites [13]. Primary peritoneal papillary serous carcinoma or primary peritoneal carcinoma is an epithelial tumor that predominantly affects women in their fifth and sixth decades of life [14].

## Diffusion pathways

Overall PM spreads within the peritoneal cavity, following the circulation and reabsorption routes of peritoneal fluid. Therefore, understanding the routes of dissemination and the dynamics of peritoneal fluid circulation is crucial for comprehending the critical signs of peritoneal carcinomatosis.

There are four dissemination routes:

- Hematogenous spread is the principal pathway for primary tumors with a high grade of malignancy.
- Contiguous spread refers to the local progression of carcinomatosis, which occurs when a large tumor crosses the serous membrane to invade adjacent organs.
- Lymphatic spread refers to the lymphatic dissemination, the lymphatic system of the greater omentum and the right side of the subphrenic lymphatic system drains into the anterior mediastinal lymphatic chain and the right lymphatic duct.
- Peritoneal surface spread refers to the location of peritoneal implants, which tend to form in anatomical regions where fluid accumulates the most, linked to gravity, intestinal peristaltic movements, and the pressure gradient caused by diaphragmatic movement during respiration, and in areas with the highest fluid reabsorption. When standing, the peritoneal fluid accumulates in the most sloping areas of the peritoneal cavity, such as the recto-vesical and paravesical recesses (Fig. 1). During exhalation, a negative pressure gradient is generated under the diaphragm, which moves the peritoneal fluid cranially through the paracolic gutters. The fluid mainly ascends along the proper paracolic gutters, which are wider than the left and communicate freely with the subdiaphragmatic and perihepatic space [15].

## CT and MRI protocols

### CT protocol

CT imaging is the first option among the primary imaging modalities to detect peritoneal implants due to its availability, fast image acquisition, spatial resolution, and multiplanar reconstructions [7, 8]. According to recent guidelines, the CT should cover the thorax, abdomen and pelvis, acquired with at least 16-slice CT equipment [7, 8]. The soft-tissue filter should be considered as a reconstruction standard, which will also include the multiplanar reconstructions in coronal and sagittal planes at 1–1.5 mm. The slice thickness should be < 0.75 mm [7, 8, 16–18]. A CT scan might be performed before and after intravenous contrast medium administration (Table 1); the portal venous phase is mandatory [7, 8]. While it is well known that the portal venous phase is regarded as the phase of choice for PC assessment, the actual effectiveness of the delayed phase is still debated [19]. Up to now, unenhanced and arterial phase are not recommended [7, 8]. CT enterography (CTE) should be considered for the study of small bowel (SB) (Table 1). With dedicated distension, the detectability of small implants might rise consistently [20].

**Table 1:** Peritoneum CT protocol

Variable	CT protocol	Timing	Required
Intravenous CM dose	0.7 gI per kg of lean body weight		Recommended
CM administration rate	3–3.5 mL/s		Recommended
Oral contrast material I. Conventional CT II. CT enterography	I. 500–800 mL of water II. 1000–1500 mL of PEG	I. 3 min before the exam II. 30–45 min before the exam	I. Optional II. Recommended
Acquisition CT phases	I Portal venous phase II. Delayed phase	I. 70 s after CM injection II. 7–8 min after CM injection	I. Mandatory II. Optional

*CM* contrast media, *PEG* polyethylene glycol

Regarding the restaging in a post-treatment setting, the main recommendations suggest following the patient with CT, with a similar protocol used for the staging setting, during the therapy or at the end, but before the debulking surgery. In doubtful cases with high suspicion of undetectable disease on CT, ^18^F-FDG PET/CT may be considered [7, 8].

## MRI protocol

Adequate bowel distension is crucial in the peritoneal MRI protocol as it enhances the accuracy and confidence in image interpretation. Before the exams, fasting for at least 4–6 h is required, and then administering 1–1.5 L of pineapple juice, as an oral contrast, in about 45–60 min, is highly suggested [7, 8, 21] (Table 2). The rationale behind the use of pineapple juice is related to the negative contrast material, which helps the radiologists differentiate the small bowel lumen from the ascites and peritoneal implants. Thus, the final results of using the oral contrast material are comparable to an MR enterography but with a negative contrast material. Additionally, administering spasmolytic agents before the exam can reduce bowel peristalsis. Suboptimal distension of the intestinal lumen may obscure thin peritoneal tumors or inflammation involving the intestinal serosa, mesentery, or adjacent peritoneum, potentially being misinterpreted as an abdominal mass [21]. MRI exams should be performed on 1.5- or 3-T MR scanners, with an external phased array coil positioning. Concerning the post-enhancement T1 sequences, the delayed phase at 3–5 min post-gadolinium administration is currently recommended for increasing sensitivity in detecting small peritoneal implants more effectively than CT or PET, while arterial and portal venous phases are not mandatory [7, 8, 22, 23]. Currently, there is no defined role for MRI in a restaging clinical setting; the use of MRI in young patients to reduce the dose exposure should always be discussed in a multidisciplinary team [7, 8].

**Table 2:** MRI protocol for the study of peritoneum

Variable	Planes	Slice Thickness/Gap	Respiration
MRE Oral CM 1000–1500 mL of pineapple juice 45–60 min before the exam			
T2 SSTSE/HASTE	• Coronal • Axial	5/0.6 mm	• Free breathing • Respiratory
STIR-DWI (TI = 160–180 ms, b = 50–1000 mm^2^/s)	• Axial • Coronal (MPR)	5/0.5 mm	• Free breathing
T1 mDixon 3D	• Axial	3/0 mm	• Breath-hold
Intravenous contrast medium			
T1 mDixon 3D FFE After 3–5 min from CM administration	• Axial • Coronal	3/0 mm	• Breath-hold • Breath-hold

*CM* contrast media, *MRE* magnetic resonance enterography, *DWI* diffusion-weighted imaging, *STIR* short T1 inversion recovery, *SSTSE* single-shot Turbo spin-echo, *HASTE* half-Fourier single-shot turbo spin-echo

## Peritoneal implants: radiological findings

The imaging findings of PM on CT and MRI vary depending on the specific histologic subtypes of the primary tumor. Peritoneal implants most commonly appear as solid lesions characterized by soft-tissue plaques, nodules, or masses with variable enhancement on CT images [1, 24]. A multiparametric imaging approach could be suggested, considering the variable PC findings.

**Fig. 2:**
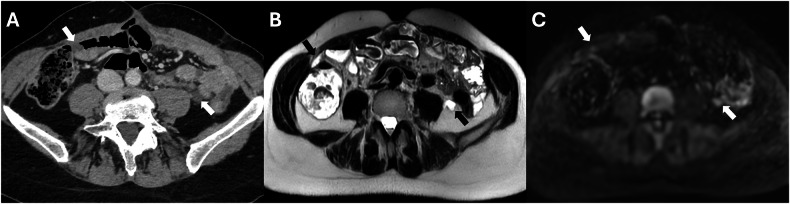
Benign peritoneum mesothelioma in a 61-year-old woman. **A** CT porta-venous phase, axial plane showed cystic implants in the right and left iliac fossa (white arrows), confirmed in MRI T2w (**B**, black arrows) and DWI (b = 800 mm^2^/s) (**C**, white arrows)
- Micronodular pattern: indicating the presence of peritoneal implants less than 5 mm in diameter, affecting the peritoneum and the mesenteric fat (Fig. 3). Infiltration of the small bowel mesentery may produce characteristic stellate patterns, which consist of increased mesenteric fat attenuation and perivascular soft-tissue thickening caused by the infiltration of the perivascular bundles [26].

In general, different patterns of lesions have been identified. In case of low burden of disease, it is more likely to have the following patterns:

- Cystic metastases are generally derived from primary mucinous carcinomas or primary peritoneal malignancies (Fig. 2) [25].

**Fig. 3:**
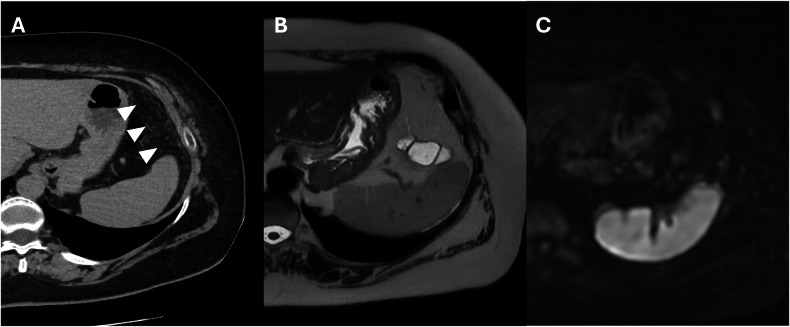
Colon adenocarcinoma with peritoneal carcinomatosis in a 74-year-old woman. **A** CT delayed phase, axial plane. Relevant diffuse implants with micronodular pattern in the left hypochondrium (arrowheads). MRI T2w and DWI (b = 800 mm^2^/s) (**B**, **C**) axial plane, no micronodular implants are visible
- The nodular pattern is characterized by a nodular lesion greater than 5 mm in diameter that might be solid or cystic, depending on the primary tumor (Fig. 4) [26].

**Fig. 4:**
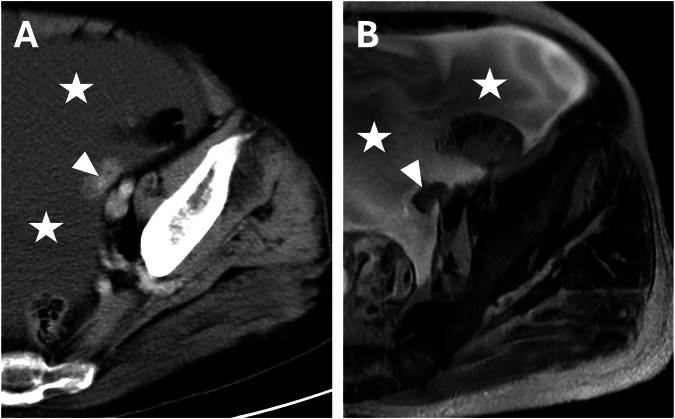
Colon adenocarcinoma with peritoneal carcinomatosis in an 81-year-old woman. CT portal-venous phase (**A**) and MRI T2w (**B**) demonstrated a nodular pattern in the left iliac fossa (arrowheads) and diffuse ascites (stars)
- Calcification: manifesting as areas of hypointensity on MRI scans, both on T1 and T2-weighted sequences and regions of hyperattenuating on CT scan (Fig. 5). Calcified peritoneal implants may suggest a primary ovarian serous cystadenocarcinoma, a primary papillary mucinous carcinoma, or as a result of chemotherapy [27].

**Fig. 5:**
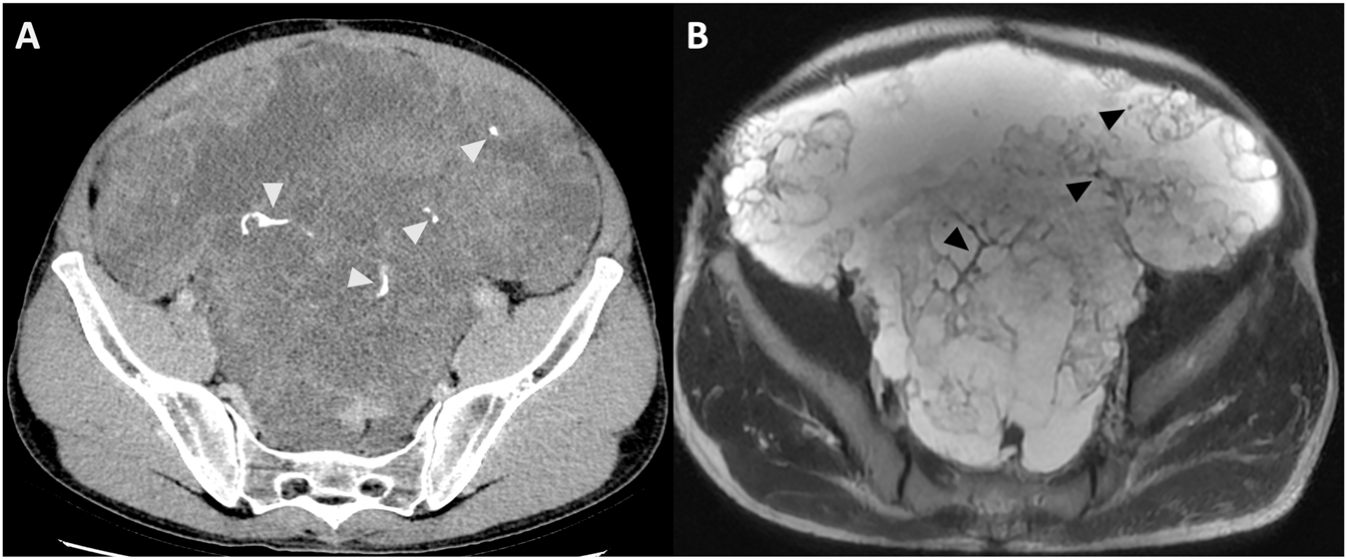
Mucinous colon cancer with peritoneal implants in a 57-year-old man. CT delayed phase (**A**) and MRI T2w (**B**) showed calcifications (arrowheads) within the mucinous implants

**Fig. 6:**
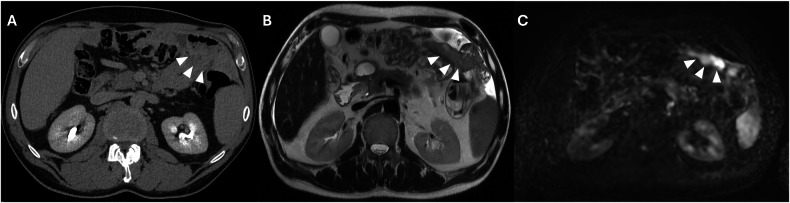
Gastric cancer with peritoneal carcinomatosis in an 81-year-old man. CT delayed phase (**A**), MRI T2w and DWI (b = 800 mm^2^/s) (**B**, **C**) axial plane showed a large implant known as a “Plaque-like” pattern (arrowheads).
- Omental cake: large plaques surrounded by reactive fibrotic tissue that results in the consolidation of omental fat, which appears stratified and causes posterior displacement of the bowel relative to the anterior abdominal wall (Fig. 7) [29].

When the disease burden is high, it is more common to have the following patterns:

- Plaque-like pattern: Plaques are irregular soft-tissue thickenings created by the confluence of multiple nodular implants typically found in the upper abdomen (Fig. 6). When plaques cover the capsular edges of intraperitoneal organs, they appear as areas of lower attenuation than the parenchyma in post-contrast scans. This creates indentations on the surface, particularly of the liver and spleen, giving a “scalloping” appearance [28].

**Fig. 7:**
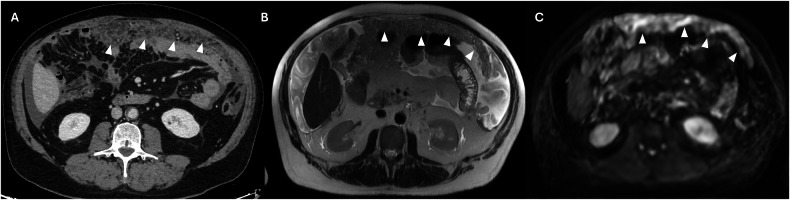
Ovarian cancer with peritoneal carcinomatosis in a 72-year-old woman. CT portal-venous phase (**A**), MRI T2w and DWI (b = 800 mm^2^/s) (arrowheads) (**B**, **C**), axial plane showed diffuse involvement of omental fat known as “Omental cake” pattern (arrowheads)
- Mass-like pattern: this pattern involves soft-tissue masses of several centimeters, resulting from the confluence of multiple nodular implants. Masses more than 10 cm in diameter are called “bulky tumors.”
- Teca pattern: This involves significant engagement of the visceral peritoneum lining the loops of the small bowel, causing thickening, distortion, and fixation of the mesentery. This culminates in intestinal obstruction, a “frozen pelvis” (Fig. 8) [30].

**Fig. 8:**
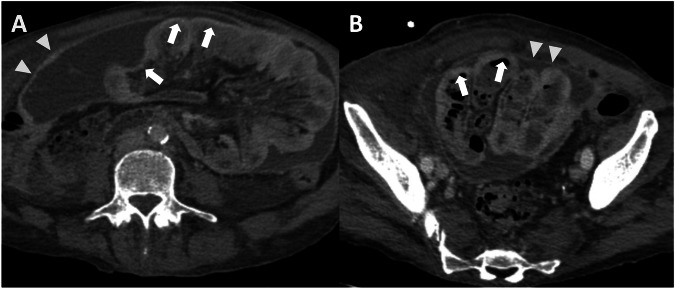
Malignant pleural mesothelioma with peritoneal implants in a 67-year-old man. CT delayed phase and portal-venous phase (**A**, **B**), axial plane. Small intestine loops were completely covered by a thickened layer of visceral peritoneum (arrowheads) known as the “Teca aspect.” Moreover, the parietal peritoneum is thickened (arrows)

## Red flags in CT and MRI

One of the significant clinical challenges is detecting small implants and giving an assessment of patients with a reduced disease burden. Then, it is essential to recognize the red-flag imaging findings that could indicate any peritoneal involvement.

The presence of ascites must be carefully monitored, as it is typically one of the earliest indicators of carcinomatosis, even if it is not specific. It can appear diffuse, loculated, or septate, often because of adhesions. One of the primary causes of ascites is the obstruction of subphrenic lymphatic vessels, leading to reduced reabsorption of peritoneal fluid. Additionally, excessive fluid production can occur due to increased capillary permeability caused by tumor cells secreting vascular permeability factors [31, 32].

**Fig. 9:**
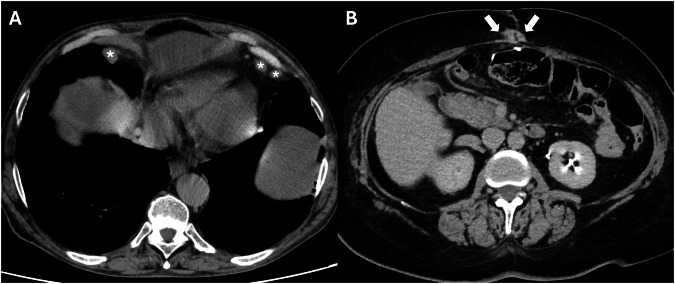
CT porta-venous phase, axial plane, showed the two most relevant red flag findings in subphrenic nodes (**A**) (stars) and subcutaneous implants in the periumbilical area are known as “Sister Mary Joseph’s nodules” (**B**) (arrows)

An unusual form of peritoneal and omental tumor involvement is the presence of subcutaneous nodules in the anterior abdominal wall. Subcutaneous metastases in the periumbilical area are known as “Sister Mary Joseph’s nodules” (Fig. 9). PM can reach the umbilicus through direct extension from the anterior peritoneum or ligamentous communications (e.g., the sickle-cell, median umbilical, or omphalomesenteric ligaments) hematogenous spread, retrograde lymphatic flow, or by the entrapment of implants at the laparoscopic entry site [33]. Metastases at the port or trocar site are highly common, and these consist of cancer recurrence in the scar tissue or at the incision wound after laparoscopy [34]. The presence of cardiophrenic angle lymph nodes is a common finding in high-risk patients and has been linked to the presence of PC (Fig. 9). Similarly to the other imaging findings, the cardiophrenic angle lymph nodes are not specific but highly suggestive [35].

## PM in the upper abdomen

The upper abdomen in peritoneal diseases is known as the supramesocolic compartment, the peritoneal space above the root of the transverse mesocolon, containing intraperitoneal organs such as the liver, spleen, and stomach. The ligaments are essential to support and connect the abdominal organs. These can divide the supramesocolic space into several subspaces, including right and left subphrenic spaces and a subhepatic space that continues into the lesser sac. The lesser and greater omentum can be included in this region; the former is stretched from the lesser curvature of the stomach to the left hepatic lobe, and the latter is a fatty membrane originating from the transverse colon and covering the entire small bowel [36]. In the upper abdomen, the hepatic and splenic surfaces are the two most frequent sites of peritoneal implants, as well as the greater omentum. These usually indent the parenchyma, resulting in a typically “scalloping” appearance of the underlying parenchyma [37].

## Pearls

The most common sites of PM are the greater omentum, the perihepatic and perisplenic regions. In the former, it usually shows a hematogenous dissemination, which might appear as micronodular, nodular or omental cake patterns [36]. While in the perihepatic and perisplenic regions, the deposits are usually solids, and these might secondarily invade the underlying parenchyma, and this assessment is generally entrusted to an imaging approach (Table 3). The absence of parenchymal invasion is suggested when a tissue plane, such as fat or ascites, exists between perihepatic metastasis and the liver parenchyma or a smooth and well-defined lesion-liver interface. Conversely, the protrusion of a perihepatic metastasis into the liver without a well-defined but ill-defined, irregular liver-lesion interface may suggest parenchyma invasion [38]. The lesser omentum is rarely involved, and when it does happen it is predominantly because of the presence of gastric cancer that results in its direct invasion [36]. In small lesions (< 1 cm), conventional CT has a sensitivity of around 25–50% due to the reduced contrast resolution [39]. While the sensitivity of MRI, with delayed post-contrast sequences, for identifying peritoneal implants < 1 cm rises to 75–80% [40]. In addition, diffusion-weighted MRI might improve the detection, especially in peritoneal reflections close to the liver and pancreas [41, 42]. However, MRI performance is low in the left hypochondrium, in which the spleen is spontaneously hyperintense on diffusion-weighted MRI, thus making perisplenic deposits less conspicuous on DWI [43].

**Table 3:** Head-to-head CT and MRI in different abdominal regions

Study	Imaging	Population	Endpoint	Performance
Upper abdomen				
Coakley et al [36]	CT	31 patients	CT accuracy implants ≤ 1 cm	Sens: 25–50% Spec: 78–96% PPV: 44–80% NPV: 78–85%
Low et al [37]	CT vs MRI	24 patients	Imaging performance in CT and MRI	Sens: 54% vs 84% Spec: 91% vs 87% Acc: 74% vs 86%
Low et al [38]	MRI	34 patients	Conventional MRI vs Conventional MRI + DWI	Sens: 52–73% vs 84–90% Spec: 90–92% vs 91% Acc: 72–81% vs 88–91%
Lower abdomen				
Choi et al [87]	CT	57 patients	CT performance	Sens: 45% Spec: 72% PPV: 46% NPV: 72%
Low et al [5]	CT and MRI	22 patients	Preoperative PCI: CT vs MRI	Sens: 55% vs 95% Spec: 86% vs 70% Acc: 63% vs 88%
Cianci et al [88]	MRI	24 patients	Conventional MRI vs Conventional MRI + DWI	Sens: 55% vs 87 Spec: 64% vs 80%
Small bowel and mesentery				
Low et al [59]	CT and MRI	164 patients	CT vs MRI Mesentery and small intestine	Mesentery Sens: 36% vs 57% Small intestine Sens: 63% vs 84%
Chua et al [57]	CT	47 patients	CT in preoperative PCI	Small intestine Acc: 21–25% Sens: 56–57% Spec: 100%
Ricke et al [21]	MRI	57 patients	Detection of peritoneal implants in ovarian cancer	Bowel and mesentery Sens: 73–77%.

*PCI* peritoneal cancer index

Regarding the subphrenic space, nodular or plaque-like thickening with enhancement along the diaphragmatic surface adjacent to the liver is the imaging sign of subphrenic involvement, often well recognizable on delayed gadolinium-enhanced MR images [24]. However, the motion artifact or susceptibility artifact at the lung bases should always be considered in MRI, and these limit the potential added value of DWI for detecting subtle tumors at this location [41].

**Fig. 10:**
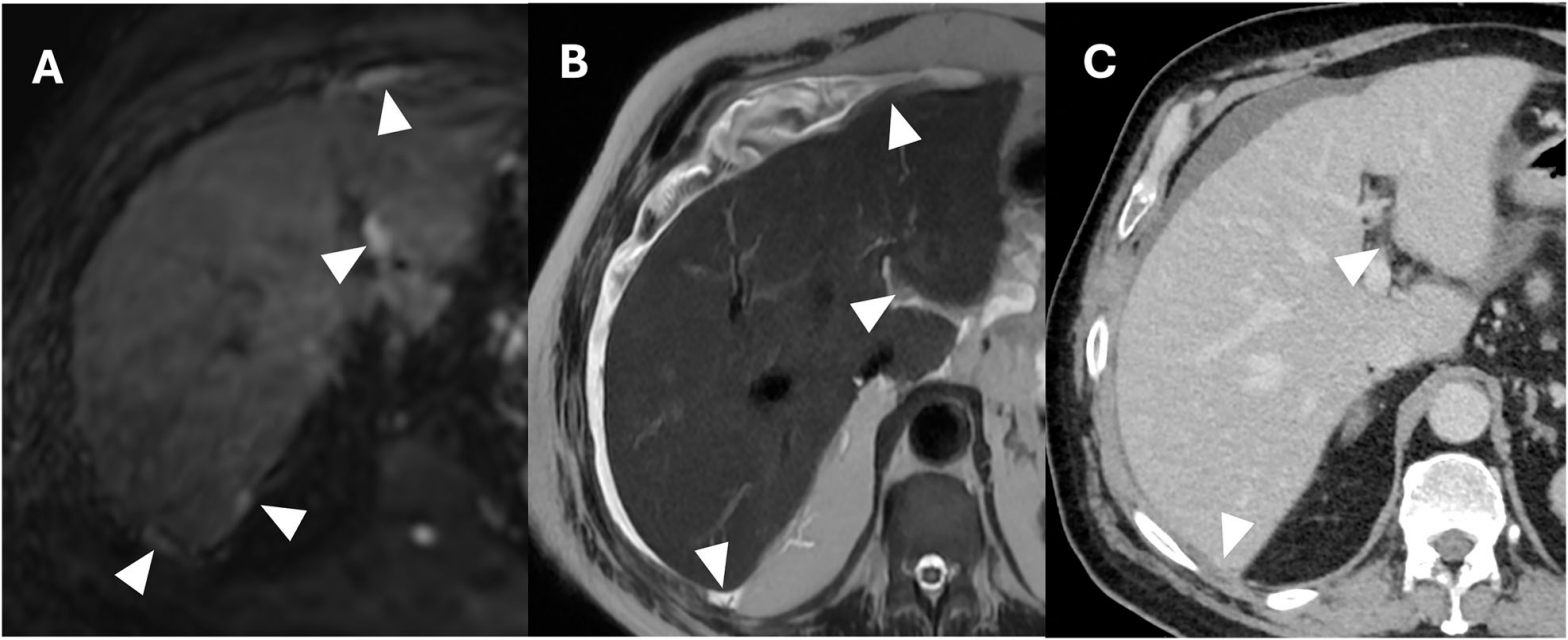
Pancreatic cancer with peritoneal carcinomatosis in a 64-year-old woman. MRI DWI (b = 800 mm^2^/s) (**A**) demonstrated four nodular implants in the right hypochondrium (arrowheads), of which only three were evident on T2w (arrowheads) (**B**) and two on CT portal-venous phase (arrowheads) (**C**)

The upper mesocolic space, including the gastrohepatic ligament and the hepatoduodenal ligament, should be evaluated to provide the surgeon with relevant information about the ligament’s involvement. The gastrohepatic ligament can be recognized by identifying the left gastric artery and the hepatoduodenal ligament by detecting both portal veins, common bile ducts, and hepatic arteries. Furthermore, the fluid distention of the lesser sac is a sign of tumor implants; other signs include thickening, nodules, and stranding [44]. Perihepatic ligaments and fissures are commonly involved with variable patterns, from diffuse soft-tissue stranding to nodular or plaque-like implants; these should always be detected and reported, considering this finding is a criterion of unresectability [44]. A direct sign of tumor infiltration of the hepatic hilum is the absence of a peri-portal fat plane due to tumor replacement [24]. Overall, DWI combined with conventional MRI seems to improve the detection of malignant deposits in the right subhepatic region compared with traditional contrast-enhanced CT, achieving a sensitivity of around 95% (Fig. 10) [41].

## PM in the lower abdomen

Since peritoneal seeding is related to fluid stasis, promoted by capillary force on surfaces and, more importantly, by gravity, it is pretty standard for peritoneal masses to be observed in the lower quadrants of the abdomen [37]. Then, tumor cells can be directed cranially via the right paracolic gutter to the Morison pouch. The left paracolic gutter is more rarely the site of disease because it is stopped superiorly by the phrenicocolic ligaments [44]. The recto-uterine space, or sac of Douglas, represents the caudal extension of the peritoneal cavity and is a frequent site of seeded lesions [45].

### Pearls

**Fig. 11:**
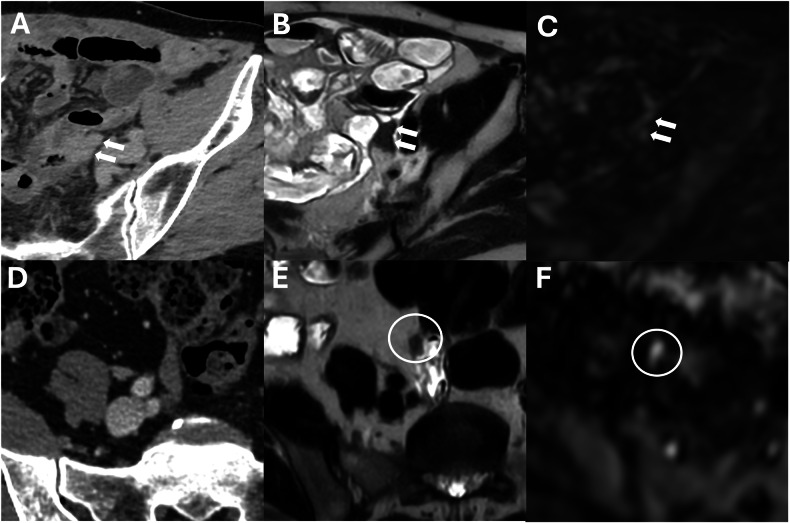
Ovarian cancer with peritoneal carcinomatosis in a 69-year-old woman (**A**–**C**). **A** CT delayed phase, axial plane showed a nodular implant in the left iliac fossa (arrows), confirmed in MRI T2w and DWI (b = 800 mm^2^/s) (arrows) (**B**, **C**). Colon adenocarcinoma with peritoneal carcinomatosis in a 64-year-old woman (**D**–**F**). MRI T2w (**E**) (circle) demonstrated a nodular implant in the distal ileum, confirmed in DWI (b = 800 mm^2^/s) (**F**) (circle), but not evident on CT portal-venous phase (**D**)

It is essential to know the histologic characterization of the primary tumor when we evaluate for possible lesions in the Douglas cavity, which is also often the site of peritoneal effusion because of its declivous anatomic location [21]. Looking at paracolic gutters, the right side is the most common site of implants due to the preferential root of peritoneal fluid, the left side is rarely involved, and the features of implants may vary according to the primary neoplasm [44]. Solid implants showed a pronounced enhancement in both CT and MRI, a low-intermediate intensity in T2 sequences, with the possibility of restricted diffusion on DWI and ADC maps (Fig. 11) (Table 3). While differentiating cystic metastases (e.g., in peritoneal mesothelioma) from fluid collections might be challenging, these exhibit low attenuation in CT and high signal in T2-weighted sequences at MRI, showing no diffusivity restriction in diffusion-weighted sequences [46] (Fig. 2). In the pelvis, miliary distribution is characteristic of epithelial ovarian carcinoma; after exfoliation of tumoral cells from primary cancer, these passively transferred through the peritoneal fluid to the peritoneum, giving lesions that are often numerous, superficial, and small in size and, therefore, difficult to remove with cytoreductive surgery [47]. The small size also poses a diagnostic problem: lesion detection for CT scans decreases dramatically for lesions < 1 cm [48]. At the same time, by using DWI-MRI, the imaging accuracy rises to around 88% [6] due to the high intensity of DWI.

In the left lower abdominal quadrant, localizations of peritoneal carcinosis are favored by the transverse course of the sigmoid mesocolon, which causes a relative stasis of fluids. For this reason, disease localizations are more frequent here than in the contralateral quadrant [37]. PMP usually originates from the right lower quadrant of the appendix, producing mucin, with CT and MRI characteristics similar to those described above for mucinous tumor localizations: a low attenuation in CT and high signal in T2-weighted sequences to MRI without diffusivity restriction in the diffusion-weighted sequences [49].

## Small bowel and mesentery

**Fig. 12:**
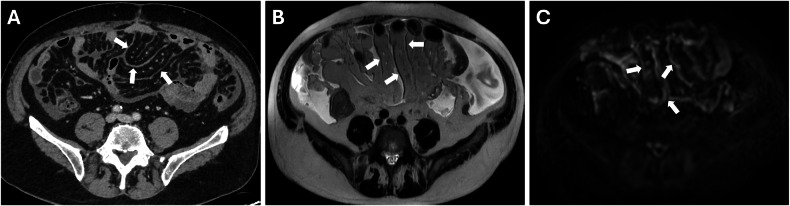
Mesenteric root infiltration in a 54-year-old man with colon cancer. CT portal venous phase (**A**), axial plane, the “misty mesentery” pattern with edema alongside the vessels (arrows). This pattern was confirmed also on MRI T2w (**B**, arrows) and on DWI (b = 800 mm^2^/s) (**C**, arrows)

The extent of peritoneal carcinosis in the small bowel (SB) and its mesentery is critical in determining the eligibility of patients for cytoreductive surgery. SB involvement frequently precludes complete resection of PM due to the substantial risk associated with performing multiple bowel anastomoses, which can lead to significant postoperative complications and the potential for short-bowel syndrome [3]. When SB, mesentery, and intestinal serosa are involved, it is necessary to report whether the extent of involvement is less than or greater than 50%, the number and location of stenosis of the small bowel, and the involvement of the colon or gastric system [22]. The serosa of the SB is generally less susceptible to cancer metastases due to its lower density of milky spots, which are small channels linking the peritoneal cavity to submesothelial lymphatic networks [50]. Consequently, tumors that infiltrate the peritoneum of the SB are presumed to be more aggressive compared to those that do not affect this area. The SB peritoneal cancer index (SB-PCI), the sum of the peritoneal cancer index (PCI) scores for the four specific areas of the SB, ranges from 0 to 12 (Fig. S1). Elias et al [3] demonstrated that an SB-PCI > 0 and the specific involvement of the lower ileum (area 12) markedly worsened the prognosis. The highest 5 years of overall survival (70%) was observed in patients without involvement of area 12 and a PCI < 15. In contrast, the 5 years of overall survival rates for patients with involvement of area 12 are significantly lower (12% for patients with PCI ≥ 15 and 15% for those with PCI < 15) [3]. The assessment of mesentery root infiltration represents a consistent radiological challenge; it might be rarely diagnosed with certainty, but it represents one of the major criteria of inoperability (Fig. 12) [51]. One of the major CT findings related to the mesenteric infiltration is known as “misty mesentery” due to the lymphatic obstruction by the tumor, resulting in edema alongside the vessels and mesenteric nodal enlargement. Alternatively, the hematogenous spread may occur, resulting in tumor emboli within the mesenteric arterial branches or serosal nodular lesions [51]. However, the overlap between malignant infiltration and non-neoplastic disease should always be considered, and in difficult cases, the biopsy might be essential.

### Enterography: CT or MRI?

There is a lack of consensus about the best option among CTE and MRE to assess the SB and mesentery involvement (Table 3), CT accuracy in detecting peritoneal lesions in SB is around 21–25% for the four small bowel regions without bowel distention [52], and thin mesenteric tumor sheets are invisible on CT, compared to the surgical finding [53]. MRE was estimated to be superior in terms of sensitivity in evaluating the intestinal tract and mesenteric involvement, especially smaller ones [54]. Despite its numerous benefits, MRI has notable limitations, such as restricted availability, high costs, extended examination durations, and extensive training for interpreting. Consequently, CTE is typically used as the primary diagnostic tool in clinical practice, while MRE is a secondary method to enhance diagnostic accuracy. When CTE results are inconclusive, the two imaging modalities are thus considered complementary.

### Pearls

Implants on the SB wall may present as nodules, masses between loops, or masses adhering to adjacent loops, potentially causing bowel obstruction (Fig. 11). The “teca pattern” is characterized by numerous small nodular implants covering the SB loops appearing as wall thickening and enhancement, restricted distensibility, distortion of SB segments with wall irregularity, or intestinal stenosis (Fig. 8) [10, 30, 55]. This thickening can cause narrowing, obstruction, and proximal loop dilation, a condition known as “frozen pelvis, “ a significant contraindication for surgery [56]. In patients with known malignancy, distinguishing between a benign condition (i.e., due to postsurgical adhesions or radiation enteritis) or malignant causes of intestinal obstruction is a radiological challenge. Russell et al [57] identified three main features that indicate malignant intestinal obstruction: (1) focal (< 10 cm) or localized (> 10 cm but less than 50% of SB) mural thickening, (2) moderate (3–10 mm) or marked (> 10 mm) peritoneal thickening, and (3) a moderate (if the degree of enhancement equals that of the liver) or marked (if the degree of enhancement equaled that of adjacent vessels) peritoneal enhancement. On the other hand, benign intestinal obstruction is indicated by the absence of an obstructive mass and a diffuse (at least 50% of SB) mural thickening [56].

Small bowel mesentery root involvement may manifest as increased attenuation or stranding of mesenteric fat, nodules, masses, or thickening with crowded vascular bundles [58].

Peritoneal malignant mesothelioma appears with a stellate infiltration of the mesentery, increased mesenteric fat attenuation, perivascular soft-tissue thickening, and vascular-bundle stiffness [59]. However, these are nonspecific signs of mesenteric involvement, which can also be present in other pathologies, such as peritoneal tuberculosis [58].

## General pitfalls

Several peritoneal diseases mimic PC, such as other secondary and primary peritoneal tumors (e.g., peritoneal lymphomatosis and peritoneal mesothelioma) or inflammatory and benign conditions (e.g., peritoneal tuberculosis, endometriosis, splenosis, and fibrosis) (Figs. S2, S3).

### Other peritoneal malignancies

Looking at secondary peritoneal malignancies, the peritoneal lymphomatosis is one of the most relevant, representing the intraperitoneal spread of lymphoma (Fig. S4). Its imaging features are similar to those of PC with diffuse thickening of peritoneal surfaces, peritoneal nodules, or masses. However, bulky homogeneous masses, diffuse retroperitoneal lymphadenopathy, hepatosplenomegaly, and hepatic or splenic nodules are helpful signs of peritoneal lymphomatosis [60, 61]. While peritoneal malignant mesothelioma is the most common primary malignant peritoneal tumor (Fig. S5). It may simulate peritoneal carcinomatosis on imaging, demonstrating diffuse peritoneal thickening, multifocal peritoneal nodules, and omental cake, with or without ascites. The diagnosis should be suggested when there is a history of asbestos exposure and concomitant pleural plaque. Additionally, calcifications and lymphadenopathy are usually absent [62, 63].

### Benign conditions

Regarding the benign conditions, peritoneal endometriosis should not be forgotten in the differential diagnosis of pelvic peritoneal lesions. Endometriosis, defined as the abnormal implantation of endometrial cells in organs other than the uterus, affects up to 45% of women of childbearing age, presenting with chronic pelvic pain and dysmenorrhea [64]. On MRI, it is easy to identify the blood component of endometriosis localizations, most frequently located in the pelvis, but in other cases also in upper abdominal regions, appear hyperintense in T1-weighted sequences and, depending on the stage of degradation, with variable intensity in T2 and has no contrast enhancement (Fig. S6). In contrast, the fibro-stromal component appears isointense in T1 and hypointense in T2 and presents enhancement even in the delayed phase, thus making it more challenging to characterize. In addition, endometriosis, like peritoneal carcinomatosis, can contribute in all quadrants of the abdomen [65]. Then, MRI is more indicated in the pelvis study because of its better sensitivity on soft tissues at this level [66], and any findings should be put in differential diagnosis with multiple local benign and malignant pathologies. A rarer benign pathology that can always occur in women is the abdominal leiomyomatosis, of uncertain origin, which may be related to uterine artery embolization and laparoscopic myomectomy [67]. It is characterized by the proliferation of smooth muscle cells, with the formation of multiple nodules that may be located primarily in the lower abdomen but may also involve the upper abdomen. The nodules appear lobulated, with well-defined margins and gradual homogeneous enhancement on both CT and MRI. On MRI, the implants may be hyperintense in T2 with intermediate hyperintensity in DWI and low signal value in ADC maps [67, 68]. However, proper differential diagnosis is difficult, both because of the rarity of abdominal leiomyomatosis and the nonspecific imaging features of the nodules. However, the patient’s remote pathological history can help in doubtful cases.

### Inflammatory conditions

Among the inflammatory diseases of the peritoneum, it is necessary to mention the peritoneal tuberculosis (Fig. S2). It is an uncommon manifestation of tuberculosis, and the imaging appearance may overlap with PC, making it difficult to differentiate between the two. However, a smooth and regular thickening of the peritoneal lining is more characteristic of peritoneal tuberculosis. Moreover, associated hypoattenuating lymphadenopathy for caseous necrosis, splenic, or lymph node calcification may suggest the diagnosis [69, 70]. Peritoneal tuberculosis has two most common patterns: the “dry/plastic” type, characterized by caseous nodules, fibrous peritoneal reactions, and dense adhesions [71], and the “fixed fibrotic” type, characterized by peritoneal masses adhering to adjacent digestive structures, which might lead to obstruction. However, specific peritoneal tuberculosis signs are the presence of mesenteric macro-nodules, enhancement and consistent thickening of the parietal peritoneum, splenomegaly and splenic calcifications, involvement of the ileocecal wall, and retroperitoneal or peri-pancreatic lymphadenopathy with a hypodense center and ring-enhancement. Furthermore, peritoneal thickening in tuberculosis tends to be smoother and more regular than PM [69, 71–73]. Another sign that can be considered typical of tuberculosis is the “omental ring” sign on CT, defined by a thin or thick rim of uniform thickness, enhancing either moderately or significantly and clearly outlining the entire or part of the omentum during the venous phase [74]. This sign can identify 85% of patients with peritoneal tuberculosis and is absent in 96% of patients with PC, making it effective in ruling out PC. One more challenging differential diagnosis is between peritoneal fibrosis and PM. The former is a condition usually occurring in a postoperative setting, also in oncological patients and patients treated with peritoneal dialysis. More literature data should be present about this head-to-head comparison. Still, the general direction is that differentiating peritoneal fibrosis from PM using conventional imaging (CT, MRI, or PET/CT) is difficult and often impossible [75].

## Multidisciplinary approach: what the surgeon needs to know

In the last few years, the prognosis has slightly improved due to a combination of hyperthermic intraperitoneal chemotherapy (HIPEC) and cytoreductive surgery (CRS) [2]. HIPEC consists of the instillation of the chemotherapeutic agent at the temperature of 41–43 °C directly into the peritoneum, aiming to achieve a high and persistent drug concentration in the tumor while limiting systemic to maximize therapeutic efficacy and minimize side effects [55]. The treatment is mainly dependent on primary cancer; peritoneum primary tumors are usually treated with an upfront surgical approach, while in the case of PC, the role of neoadjuvant or adjuvant chemotherapy is relevant [76].

In identifying patients who could benefit from surgical resection, imaging has the leading role of detecting the two most essential criteria for unresectability and describing disease burden in terms of peritoneal cancer index (PCI) (Fig. 12) [77]. Historically, PCI was introduced as a quantitative surgical score providing important patient prognosis information [78]. It consists of surgical exploration of 13 abdominal regions, determining the extension of the cancer (9 superficial quadrants and 4 intestinal segments). In each of these regions is given a score from L0 to L3: L0 is the absence of tumor; L1 means lesion size under 0.5 cm; L2 is a lesion between 0.5 and 5 cm; L3 is a tumor larger than 5 cm. Minimum score is 0, while the maximum score is 39. From a predictive point of view, a PCI score lower or equal to 10 means a 5-year survival rate of 50%, PCI between 11 and 20 means a survival rate of 20%, and PCI over 20 means a survival rate under 10%. PCI over 20 contraindicates cytoreductive surgery [79]. Thus, the radiological challenge was to predict a radiological PCI to provide some relevant information regarding prognosis and resectability (Fig. S7). Some comparative studies demonstrated intermediate results in terms of sensitivity and specificity of both CT- and MRI-PCI [5]; more specifically, the assessment of general CT-PCI achieved a sensitivity of 76% and specificity of 69% [3]. On one hand, MRI is more accurate in identifying small implants and investigating the status of the small intestine [5, 80, 81]. On the other hand, CT has several advantages, such as three-dimensional visualization, spatial resolution, and high availability, compared to MRI, which has high costs, long scanning time, reduced availability, potential artifacts due to patients’ movement, magnetic susceptibility, reduced inter-reader agreement, and low spatial resolution [1]. Despite the difficulties in accurately predicting PCI, it is recommended to report it in the radiology report to provide the surgeon with as much information as possible regarding the disease burden. To date, there are no universal criteria for inoperability; much depends on the decisions provided by the multidisciplinary meetings, and the experience of the surgical team [82]. There are a few points that should always be discussed: first, the quality of life for the patient after surgery, second, the possibility of getting to an R0, and finally, the extent of disease. All these points depend on the sites of disease; in fact, some are difficult to attack surgically, among them we have extra- and retroperitoneal lesions, involvement of the small intestine, hepatic hilum, mesentery, and pelvic structures such as the bladder [83].

Regarding the restaging assessment, while there is the completeness of cytoreduction score (CCR) a validated surgical score for reassessment of response to therapy [84], from the imaging perspective the recent guidelines suggest to re-assess the patients with similar CT protocol to staging setting. Considering the ^18^F-FDG PET/CT only in doubtful cases, as a high suspicion of persistent but undetectable disease on CT scan [8].

Regarding post-treatment residual disease, only few data are available in the literature; however, the emerging finding concerns the inability of CT to differentiate fibrosis and necrosis from residual disease [85]. Recently, a simplified peritoneal tumor index (S-PCI) score was tested as a CT predictor of treatment response, but no significant results were obtained in the correlation between S-PCI and pathologic chemotherapy response score [85]. Even in the new international guidelines, there is no clear indication regarding residual disease or PM restaging [7, 8]. However, it is essential to consider that in many malignancies the term of restaging may coincide with the preoperative term; in fact, neoadjuvant chemotherapy is almost always recommended in cases of peritoneal involvement.

## What’s in the future?

Beyond the conventional imaging methods, new modalities have been acquiring promising results in diagnosing and assessing patients with PM, including PET/CT with fibroblast activation protein inhibitor (FAPI), PET/MRI, whole-body MRI, and spectral CT. FAPI-PET might be seen as a target imaging, due to the fibroblast activation protein inhibitor (FAPI), a radiopharmaceutical labeled with either ^68^Ga (gallium-68) or ^18^F (Fluorine-18), targeting the fibroblast activation protein (FAP), overexpressed in cancer-associated fibroblasts (CAF) and rarely expressed in normal tissues [86]. By using FAPI-PET, it could be possible to identify the peritumoral stroma and support matrix, mainly in epithelial tumors, and in very small lesions (> 2 mm) [87]. In comparison with conventional FDG-PET, the FAPI-PET could be superior in detecting small micronodular lesions [88–90], also covering the gap for low FDG-avidity neoplasms (e.g., mucinous adenocarcinoma), having a higher tumor-to-background ratio than FDG-PET due to low intestinal and hepatic uptake [91]. Until now, FAPI-PET has been used for clinical research studies, but more large-scale comparative studies with long-term follow-up are required. False-positive findings are not rare in patients with inflammatory diseases (gastritis, abscesses), granulomatous diseases, or conditions where a fibrotic reaction is present (e.g., myelofibrosis and liver cirrhosis) [92]. Remaining in functional imaging, the proposal is to integrate PET and MRI, which could enhance peritoneal implant detection by synergistically providing anatomical, functional, and metabolic information while reducing radiation exposure. Furtado et al [93] found that PET/MRI might be more effective than PET alone and MRI or in combination with CT, changing the therapeutic management in 26% (5/19) of patients.

New perspectives are emerging about the usefulness of whole-body MRI, mainly in the detection of extra-peritoneal lesions, such as enlarged lymph nodes, pleural lesions, and small liver metastases [7]. Having a comprehensive view of disease burden is essential in all patients who are candidates for HIPEC treatment, and then design the therapeutic scheme tailored to the patient [94]. The recommended sequences for the whole-body MRI are the axial T2 with reduced slice thickness (≤ 4 mm), DWI with b50 and b1000 s/mm^2^, and coronal/axial T1 post-contrast (3–5 min). The use of negative oral contrast medium and the antispasmodic agent is recommended but optional [7].

Finally, spectral CT scanners could be promising in PM, thanks to their technologies based on dual-energy or multi-energy technologies. The new photon-counting spectral CT (SPCCT) offers the highest spatial resolution, a high contrast-to-noise ratio (CNR), reduced radiation dose, and enables more than two types of multi-contrast imaging [95]. These data can be converted into various types of spectral imaging, such as iodine density imaging, virtual non-contrast imaging (VMI), virtual monoenergetic imaging, and Z-effective imaging. VMI allows for low-energy reconstructions (40–50 keV) that enhance the visualization of intravascular contrast, aiding in better differentiation of small and vascularized lesions with a lower dose of contrast agent and reducing the need for costly and lengthy examinations such as MRI. To date only limited data are available in the literature regarding the applicability of spectral maps to peritoneal malignancies, however, the emerging data suggest that VMI at low energy levels produced substantially higher CNR and SNR values with superior lesion detection rate [96] and SPCCT (with a dual-contrast protocol between the peritoneum and blood vessels) has higher sensitivity (69%) and specificity (100%) compared to conventional CT for small lesions (< 5 mm) [97].

Despite the promising data emerging from the new imaging methods, validation in multicenter studies is needed for their utility in clinical practice to be approved.

## Conclusions

The diagnosis, preoperative assessment, and therapeutic workflow of peritoneal malignancies mainly depend on a multimodality approach. CT scan remains the first imaging method due to its availability, robustness, and panoramic evaluation. Overall, CT has several consistent limitations concerning identifying small implants in critical regions. MRI could support the radiologist’s ability to provide a more accurate preoperative assessment, especially in evaluating essential areas. Becoming aware of the mismatch between radiological and surgical PCI and having a multimodality imaging approach is helpful to reduce the standard CT and MRI pitfalls leading to over- or underestimation of peritoneal involvement.

## Supplementary information

ELECTRONIC SUPPLEMENTARY MATERIAL

## Data Availability

All data are available from the corresponding author.

## Abbreviations

HIPEC: Hyperthermic intraperitoneal chemotherapy
PC: Peritoneal carcinomatosis
PCI: Peritoneal cancer index
PM: Peritoneal malignancies
PMP: Pseudomyxoma peritonei
SB: Small bowel

## References

[1] Iafrate F, Ciolina M, Sammartino P et al (2012) Peritoneal carcinomatosis: imaging with 64-MDCT and 3T MRI with diffusion-weighted imaging. Abdom Imaging 37:616–627 21972153 10.1007/s00261-011-9804-z

[2] Spiliotis J, Halkia E, de Bree E (2016) Treatment of peritoneal surface malignancies with hyperthermic intraperitoneal chemotherapy—current perspectives. Curr Oncol 23:e266–e275 27330364 10.3747/co.23.2831 PMC4900847

[3] Elias D, Mariani A, Cloutier AS et al (2014) Modified selection criteria for complete cytoreductive surgery plus HIPEC based on peritoneal cancer index and small bowel involvement for peritoneal carcinomatosis of colorectal origin. Eur J Surg Oncol 40:1467–1473 25086990 10.1016/j.ejso.2014.06.006

[4] Di Giorgio A, Naticchioni E, Biacchi D et al (2008) Cytoreductive surgery (peritonectomy procedures) combined with hyperthermic intraperitoneal chemotherapy (HIPEC) in the treatment of diffuse peritoneal carcinomatosis from ovarian cancer. Cancer 113:315–325 18473354 10.1002/cncr.23553

[5] Low RN, Barone RM, Lucero J (2015) Comparison of MRI and CT for predicting the Peritoneal Cancer Index (PCI) preoperatively in patients being considered for cytoreductive surgical procedures. Ann Surg Oncol 22:1708–1715 25201499 10.1245/s10434-014-4041-7

[6] van’t Sant I, Engbersen MP, Bhairosing PA et al (2020) Diagnostic performance of imaging for the detection of peritoneal metastases: a meta-analysis. Eur Radiol 30:3101–3112 32065287 10.1007/s00330-019-06524-x

[7] Vandecaveye V, Rousset P, Nougaret S et al (2024) Imaging of peritoneal metastases of ovarian and colorectal cancer: joint recommendations of ESGAR, ESUR, PSOGI, and EANM. Eur Radiol. 10.1007/s00330-024-11124-5 10.1007/s00330-024-11124-5 PMC12021955 39499302

[8] Rizzo S, Avesani G, Panico C et al (2025) Ovarian cancer staging and follow-up: updated guidelines from the European Society of Urogenital Radiology female pelvic imaging working group. Eur Radiol. 10.1007/s00330-024-11300-7 10.1007/s00330-024-11300-7 PMC12165971 39798005

[9] Patel RR, Planche K (2013) Applied peritoneal anatomy. Clin Radiol 68:509–520 23149392 10.1016/j.crad.2012.06.135

[10] Levy AD, Shaw JC, Sobin LH (2009) Secondary tumors and tumorlike lesions of the peritoneal cavity: imaging features with pathologic correlation. Radiographics 29:347–373 19325052 10.1148/rg.292085189

[11] Tsoukalas N, Tsapakidis K, Tolia M et al (2024) Pseudomyxoma peritonei: a challenging clinical diagnosis. Case report and review of the literature. Cancer Diagn Progn 4:198–203 38434922 10.21873/cdp.10308 PMC10905292

[12] Chun CP, Song LX, Zhang HP et al (2023) Malignant peritoneal mesothelioma. Am J Med Sci 365:99–103 35940275 10.1016/j.amjms.2022.07.008

[13] Sudanthiram R, Vemulapalli C, Raja A, Santhosam R, Joseph S (2012) Malignant peritoneal mesothelioma presenting as a complex omental lesion. Radiol Case Rep 7:539 27326274 10.2484/rcr.v7i2.539 PMC4899850

[14] Miguez González J, Calaf Forn F, Pelegrí Martínez L et al (2023) Primary and secondary tumors of the peritoneum: key imaging features and differential diagnosis with surgical and pathological correlation. Insights Imaging 14:115 37395913 10.1186/s13244-023-01417-6 PMC10317950

[15] Meyers MA (1973) Distribution of intra-abdominal malignant seeding: dependency on dynamics of flow of ascitic fluid. Am J Roentgenol Radium Ther Nucl Med 119:198–206 4744725 10.2214/ajr.119.1.198

[16] Panagiotopoulou PB, Courcoutsakis N, Tentes A, Prassopoulos P (2021) CT imaging of peritoneal carcinomatosis with surgical correlation: a pictorial review. Insights Imaging 12:168 34767065 10.1186/s13244-021-01110-6 PMC8589944

[17] Jensen CT, Vicens-Rodriguez RA, Wagner-Bartak NA et al (2015) Multidetector CT detection of peritoneal metastases: evaluation of sensitivity between standard 2.5 mm axial imaging and maximum-intensity-projection (MIP) reconstructions. Abdom Imaging 40:2167–2172 25666971 10.1007/s00261-015-0370-7

[18] Delgado-Barriga K, Medina C, Gomez-Quiles L, Marco-Domenech SF, Escrig J, Llueca A (2022) CT enterography for preoperative evaluation of peritoneal carcinomatosis index in advanced ovarian cancer. J Clin Med 11:476 10.3390/jcm11030476 PMC8836697 35159927

[19] Rodolfino E, Devicienti E, Miccò M et al (2016) Diagnostic accuracy of MDCT in the evaluation of patients with peritoneal carcinomatosis from ovarian cancer: is delayed enhanced phase really effective? Eur Rev Med Pharmacol Sci 20:4426–4434 27874958

[20] Courcoutsakis N, Tentes AA, Astrinakis E, Zezos P, Prassopoulos P (2013) CT-Enteroclysis in the preoperative assessment of the small-bowel involvement in patients with peritoneal carcinomatosis, candidates for cytoreductive surgery and hyperthermic intraperitoneal chemotherapy. Abdom Imaging 38:56–63 22410875 10.1007/s00261-012-9869-3

[21] van’t Sant I, van Eden WJ, Engbersen MP et al (2019) Diffusion-weighted MRI assessment of the peritoneal cancer index before cytoreductive surgery. Br J Surg 106:491–498 30353920 10.1002/bjs.10989

[22] Low RN, Barone RM, Gurney JM, Muller WD (2008) Mucinous appendiceal neoplasms: preoperative MR staging and classification compared with surgical and histopathologic findings. AJR Am J Roentgenol 190:656–665 18287436 10.2214/AJR.07.2018

[23] Ricke J, Sehouli J, Hach C, Hänninen EL, Lichtenegger W, Felix R (2003) Prospective evaluation of contrast-enhanced MRI in the depiction of peritoneal spread in primary or recurrent ovarian cancer. Eur Radiol 13:943–949 12695813 10.1007/s00330-002-1712-8

[24] Low RN (2007) MR imaging of the peritoneal spread of malignancy. Abdom Imaging 32:267–283 17334873 10.1007/s00261-007-9210-8

[25] Low RN, Barone RM, Rousset P (2021) Peritoneal MRI in patients undergoing cytoreductive surgery and HIPEC: history, clinical applications, and implementation. Eur J Surg Oncol 47:65–74 30852063 10.1016/j.ejso.2019.02.030

[26] Kawamoto S, Urban BA, Fishman EK (1999) CT of epithelial ovarian tumors. Radiographics 19:S85–S102 10.1148/radiographics.19.suppl_1.g99oc10s85 10517447

[27] Agarwal A, Yeh BM, Breiman RS, Qayyum A, Coakley FV (2004) Peritoneal calcification: causes and distinguishing features on CT. AJR Am J Roentgenol 182:441–445 14736678 10.2214/ajr.182.2.1820441

[28] Walkey MM, Friedman AC, Sohotra P, Radecki PD (1988) CT manifestations of peritoneal carcinomatosis. AJR Am J Roentgenol 150:1035–1041 3258703 10.2214/ajr.150.5.1035

[29] Han Q, Ganesh H, DiSantis DJ (2016) Omental cake. Abdom Radiol (NY) 41:2080–2081 27259336 10.1007/s00261-016-0795-7

[30] Woodward PJ, Hosseinzadeh K, Saenger JS (2004) From the archives of the AFIP: radiologic staging of ovarian carcinoma with pathologic correlation. Radiographics 24:225–246 14730048 10.1148/rg.241035178

[31] Saif MW, Siddiqui IA, Sohail MA (2009) Management of ascites due to gastrointestinal malignancy. Ann Saudi Med 29:369–377 19700895 10.4103/0256-4947.55167 PMC3290049

[32] Chang DK, Kim JW, Kim BK et al (2005) Clinical significance of CT-defined minimal ascites in patients with gastric cancer. World J Gastroenterol 11:6587–6592 16425349 10.3748/wjg.v11.i42.6587 PMC4355749

[33] DeBardeleben J, Cohen M, Rodgers SK (2017) Peritoneal carcinomatosis presenting as a Sister Mary Joseph nodule. Ultrasound Q 33:300–302 29112635 10.1097/RUQ.0000000000000314

[34] Macias V, Baiotto B, Pardo J, Munoz F, Gabriele P (2003) Laparotomy wound recurrence of endometrial carcinoma. Gynecol Oncol 91:429–434 14599879 10.1016/j.ygyno.2003.07.001

[35] Jeune F, Brouquet A, Caramella C et al (2016) Cardiophrenic angle lymph node is an indicator of metastatic spread but not specifically peritoneal carcinomatosis in colorectal cancer patients: results of a prospective validation study in 91 patients. Eur J Surg Oncol 42:861–868 27010101 10.1016/j.ejso.2016.02.256

[36] Young JJ, Pahwa A, Patel M et al (2019) Ligaments and lymphatic pathways in gastric adenocarcinoma. Radiographics 39:668–689 30951438 10.1148/rg.2019180113

[37] Raptopoulos V, Gourtsoyiannis N (2001) Peritoneal carcinomatosis. Eur Radiol 11:2195–2206 11702160 10.1007/s003300100998

[38] Akin O, Sala E, Moskowitz CS et al (2008) Perihepatic metastases from ovarian cancer: sensitivity and specificity of CT for the detection of metastases with and those without liver parenchymal invasion. Radiology 248:511–517 18519739 10.1148/radiol.2482070371

[39] Coakley FV, Choi PH, Gougoutas CA et al (2002) Peritoneal metastases: detection with spiral CT in patients with ovarian cancer. Radiology 223:495–499 11997559 10.1148/radiol.2232011081

[40] Low RN, Barone RM, Lacey C, Sigeti JS, Alzate GD, Sebrechts CP (1997) Peritoneal tumor: MR imaging with dilute oral barium and intravenous gadolinium-containing contrast agents compared with unenhanced MR imaging and CT. Radiology 204:513–520 9240546 10.1148/radiology.204.2.9240546

[41] Low RN, Sebrechts CP, Barone RM, Muller W (2009) Diffusion-weighted MRI of peritoneal tumors: comparison with conventional MRI and surgical and histopathologic findings—a feasibility study. AJR Am J Roentgenol 193:461–470 19620444 10.2214/AJR.08.1753

[42] Low RN, Gurney J (2007) Diffusion-weighted MRI (DWI) in the oncology patient: value of breathhold DWI compared to unenhanced and gadolinium-enhanced MRI. J Magn Reson Imaging 25:848–858 17335018 10.1002/jmri.20864

[43] Berthelin MA, Barral M, Eveno C et al (2019) Preoperative assessment of splenic involvement in patients with peritoneal carcinomatosis with CT and MR imaging. Eur J Radiol 110:60–65 30599874 10.1016/j.ejrad.2018.11.022

[44] Nougaret S, Addley HC, Colombo PE et al (2012) Ovarian carcinomatosis: how the radiologist can help plan the surgical approach. Radiographics 32:1775–1800 23065169 10.1148/rg.326125511

[45] Heller DS (2016) Lesions of the pouch of Douglas: a review. J Minim Invasive Gynecol 23:28–33 26304722 10.1016/j.jmig.2015.08.878

[46] Yacoub JH, Clark JA, Paal EE, Manning MA (2021) Approach to cystic lesions in the abdomen and pelvis, with radiologic-pathologic correlation. Radiographics 41:1368–1386 34469214 10.1148/rg.2021200207 PMC8415047

[47] van Baal JOAM, van Noorden CJF, Nieuwland R et al (2018) Development of peritoneal carcinomatosis in epithelial ovarian cancer: a review. J Histochem Cytochem 66:67–83 29164988 10.1369/0022155417742897 PMC5794203

[48] Lee EYP, An H, Tse KY, Khong PL (2020) Molecular imaging of peritoneal carcinomatosis in ovarian carcinoma. AJR Am J Roentgenol 215:305–312 32551907 10.2214/AJR.19.22621

[49] Menassel B, Duclos A, Passot G et al (2016) Preoperative CT and MRI prediction of non-resectability in patients treated for pseudomyxoma peritonei from mucinous appendiceal neoplasms. Eur J Surg Oncol 42:558–566 26856956 10.1016/j.ejso.2016.01.005

[50] Yonemura Y, Kawamura T, Bandou E, Tsukiyama G, Endou Y, Miura M (2007) The natural history of free cancer cells in the peritoneal cavity. Recent Results Cancer Res 169:11–23 17506246 10.1007/978-3-540-30760-0_2

[51] Taffel MT, Khati NJ, Hai N, Yaghmai V, Nikolaidis P (2014) De-misty-fying the mesentery: an algorithmic approach to neoplastic and non-neoplastic mesenteric abnormalities. Abdom Imaging 39:892–907 24633598 10.1007/s00261-014-0113-1

[52] Chua TC, Al-Zahrani A, Saxena A et al (2011) Determining the association between preoperative computed tomography findings and postoperative outcomes after cytoreductive surgery and perioperative intraperitoneal chemotherapy for pseudomyxoma peritonei. Ann Surg Oncol 18:1582–1589 21207170 10.1245/s10434-010-1492-3

[53] Dromain C, Leboulleux S, Auperin A et al (2008) Staging of peritoneal carcinomatosis: enhanced CT vs. PET/CT. Abdom Imaging 33:87–93 17632751 10.1007/s00261-007-9211-7

[54] Low RN, Semelka RC, Worawattanakul S, Alzate GD (2000) Extrahepatic abdominal imaging in patients with malignancy: comparison of MR imaging and helical CT in 164 patients. J Magn Reson Imaging 12:269–277 10931590 10.1002/1522-2586(200008)12:2<269::aid-jmri9>3.0.co;2-g

[55] Reginelli A, Giacobbe G, Del Canto MT et al (2023) Peritoneal carcinosis: what the radiologist needs to know. Diagnostics (Basel) 13:1974 10.3390/diagnostics13111974 PMC10252296 37296826

[56] Pannu HK, Bristow RE, Montz FJ, Fishman EK (2003) Multidetector CT of peritoneal carcinomatosis from ovarian cancer. Radiographics 23:687–701 12740470 10.1148/rg.233025105

[57] Low RN, Chen SC, Barone R (2003) Distinguishing benign from malignant bowel obstruction in patients with malignancy: findings at MR imaging. Radiology 228:157–165 12832579 10.1148/radiol.2281020728

[58] Pang Y, Li Y, Xu D, Sun X, Hou D (2023) Differentiating peritoneal tuberculosis and peritoneal carcinomatosis based on a machine learning model with CT: a multicentre study. Abdom Radiol (NY) 48:1545–1553 36912909 10.1007/s00261-022-03749-1 PMC10009348

[59] Puvaneswary M, Chen S, Proietto T (2002) Peritoneal mesothelioma: CT and MRI findings. Australas Radiol 46:91–96 11966596 10.1046/j.1440-1673.2001.01002.x

[60] Kim Y, Cho O, Song S, Lee H, Rhim H, Koh B (1998) Peritoneal lymphomatosis: CT findings. Abdom Imaging 23:87–90 9437071 10.1007/s002619900292

[61] Cabral FC, Krajewski KM, Kim KW, Ramaiya NH, Jagannathan JP (2013) Peritoneal lymphomatosis: CT and PET/CT findings and how to differentiate between carcinomatosis and sarcomatosis. Cancer Imaging 13:162–170 23598428 10.1102/1470-7330.2013.0018 PMC3629893

[62] Park JY, Kim KW, Kwon HJ et al (2008) Peritoneal mesotheliomas: clinicopathologic features, CT findings, and differential diagnosis. AJR Am J Roentgenol 191:814–825 18716115 10.2214/AJR.07.3628

[63] Jeong YJ, Kim S, Kwak SW et al (2008) Neoplastic and nonneoplastic conditions of serosal membrane origin: CT findings. Radiographics 28:801–817 18480485 10.1148/rg.283075082

[64] Rolla E (2019) Endometriosis: advances and controversies in classification, pathogenesis, diagnosis, and treatment. F1000Res 8:F1000 10.12688/f1000research.14817.1 PMC6480968 31069056

[65] Gangadhar K, Mahajan A, Sable N, Bhargava P (2017) Magnetic resonance imaging of pelvic masses: a compartmental approach. Semin Ultrasound CT MR 38:213–230 28705369 10.1053/j.sult.2016.11.004

[66] Nougaret S, Nikolovski I, Paroder V et al (2019) MRI of tumors and tumor mimics in the female pelvis: anatomic pelvic space-based approach. Radiographics 39:1205–1229 31283453 10.1148/rg.2019180173 PMC6677288

[67] Woo J, Choi SY, Kim HK, Lee JE, Lee MH, Lim S (2023) Extremely rare CT and MRI findings of peritoneal leiomyoma mimicking hepatic mass: a case report. J Korean Soc Radiol 84:946–951 37559801 10.3348/jksr.2022.0032 PMC10407062

[68] Tanaka YO, Tsunoda H, Sugano M et al (2009) MR and CT findings of leiomyomatosis peritonealis disseminata with emphasis on assisted reproductive technology as a risk factor. Br J Radiol 82:e44–e47 19211902 10.1259/bjr/74052430

[69] Akhan O, Pringot J (2002) Imaging of abdominal tuberculosis. Eur Radiol 12:312–323 11870428 10.1007/s003300100994

[70] Ha HK, Jung JI, Lee MS et al (1996) CT differentiation of tuberculous peritonitis and peritoneal carcinomatosis. AJR Am J Roentgenol 167:743–748 8751693 10.2214/ajr.167.3.8751693

[71] Prapruttam D, Hedgire SS, Mani SE, Chandramohan A, Shyamkumar NK, Harisinghani M (2014) Tuberculosis—the great mimicker. Semin Ultrasound CT MR 35:195–214 24929261 10.1053/j.sult.2014.02.002

[72] Yilmaz T, Sever A, Gür S, Killi RM, Elmas N (2002) CT findings of abdominal tuberculosis in 12 patients. Comput Med Imaging Graph 26:321–325 12204236 10.1016/s0895-6111(02)00029-0

[73] Vázquez Muñoz E, Gómez-Cerezo J, Atienza Saura M, Vázquez Rodriguez JJ (2004) Computed tomography findings of peritoneal tuberculosis: systematic review of seven patients diagnosed in 6 years (1996–2001). Clin Imaging 28:340–343 15471665 10.1016/S0899-7071(03)00317-6

[74] Ramanan RV, Venu V (2019) Differentiation of peritoneal tuberculosis from peritoneal carcinomatosis by the Omental Rim sign. A new sign on contrast enhanced multidetector computed tomography. Eur J Radiol 113:124–134 30927936 10.1016/j.ejrad.2019.02.019

[75] Hong JH, Song SH, Kim SE, Lee JK, Lee NW, Lee KW (2011) Diffuse intraabdominal fibrosis and inflammation mimicking peritoneal carcinomatosis recurred after surgery for borderline ovarian tumor misdiagnosed by 18F-fluorodeoxyglucose-positron emission tomography. Eur J Gynaecol Oncol 32:231–233 21614927

[76] Colbourne JRM, Alhayo ST, Nandakumar B et al (2022) Cost-effectiveness of iterative cytoreductive surgery and hyperthermic intraperitoneal chemotherapy for the treatment of peritoneal carcinomatosis. In Vivo 36:1527–1533 35478133 10.21873/invivo.12863 PMC9087096

[77] Caruso D, Sammartino P, Polici M et al (2024) Imaging of peritoneal surface malignancies. J Surg Oncol. 10.1002/jso.27979 10.1002/jso.27979 PMC11826024 39508563

[78] Sugarbaker PH (1995) Peritonectomy procedures. Ann Surg 221:29–42 7826158 10.1097/00000658-199501000-00004 PMC1234492

[79] Duhr CD, Kenn W, Kickuth R et al (2011) Optimizing of preoperative computed tomography for diagnosis in patients with peritoneal carcinomatosis. World J Surg Oncol 9:171 22188796 10.1186/1477-7819-9-171 PMC3280941

[80] Schmidt S, Meuli RA, Achtari C, Prior JO (2015) Peritoneal carcinomatosis in primary ovarian cancer staging: comparison between MDCT, MRI, and 18F-FDG PET/CT. Clin Nucl Med 40:371–377 25783507 10.1097/RLU.0000000000000768

[81] Engbersen MP, Van’ T Sant I, Lok C et al (2019) MRI with diffusion-weighted imaging to predict feasibility of complete cytoreduction with the peritoneal cancer index (PCI) in advanced stage ovarian cancer patients. Eur J Radiol 114:146–151 31005166 10.1016/j.ejrad.2019.03.007

[82] Dohan A, Hobeika C, Najah H, Pocard M, Rousset P, Eveno C (2018) Preoperative assessment of peritoneal carcinomatosis of colorectal origin. J Visc Surg 155:293–303 29602696 10.1016/j.jviscsurg.2018.01.002

[83] Miceli V, Gennarini M, Tomao F et al (2023) Imaging of peritoneal carcinomatosis in advanced ovarian cancer: CT, MRI, radiomic features and resectability criteria. Cancers (Basel) 15:5827 10.3390/cancers15245827 PMC10741537 38136373

[84] Jacquet P, Sugarbaker PH (1996) Clinical research methodologies in diagnosis and staging of patients with peritoneal carcinomatosis. Cancer Treat Res 82:359–374 8849962 10.1007/978-1-4613-1247-5_23

[85] Roseland ME, Ma T, Shampain KL et al (2024) Neoadjuvant chemotherapy for high-grade serous ovarian cancer: radiologic-pathologic correlation of response assessment and predictors of progression. Abdom Radiol (NY) 49:2040–2048 38478037 10.1007/s00261-024-04215-w

[86] Gilardi L, Airo Farulla LS, Demirci E, Clerici I, Omodeo Sale E, Ceci F (2022) Imaging cancer-associated fibroblasts (CAFs) with FAPi PET. Biomedicines 10:523 10.3390/biomedicines10030523 PMC8945705 35327325

[87] Chen H, Zhao L, Ruan D et al (2021) Usefulness of [^68^Ga]Ga-DOTA-FAPI-04 PET/CT in patients presenting with inconclusive [^18^F]FDG PET/CT findings. Eur J Nucl Med Mol Imaging 48:73–86 32588089 10.1007/s00259-020-04940-6

[88] Capobianco A, Cottone L, Monno A, Manfredi AA, Rovere-Querini P (2017) The peritoneum: healing, immunity, and diseases. J Pathol 243:137–147 28722107 10.1002/path.4942

[89] Mori Y, Dendl K, Cardinale J, Kratochwil C, Giesel FL, Haberkorn U (2023) FAPI PET: fibroblast activation protein inhibitor use in oncologic and nononcologic disease. Radiology 306:e220749 36594838 10.1148/radiol.220749

[90] Zhao L, Pang Y, Luo Z et al (2021) Role of [^68^Ga]Ga-DOTA-FAPI-04 PET/CT in the evaluation of peritoneal carcinomatosis and comparison with [^18^F]-FDG PET/CT. Eur J Nucl Med Mol Imaging 48:1944–1955 33415432 10.1007/s00259-020-05146-6

[91] Guzel Y, Kaplan I (2023) Comparison of ^68^GA-FAPI-04 PET/CT and ^18^F-FDG PET/CT findings in peritonitis carcinomatosa cases. Hell J Nucl Med 26:26–34 37031420 10.1967/s002449912553

[92] Pang Y, Zhao L, Luo Z et al (2021) Comparison of ^68^Ga-FAPI and ^18^F-FDG uptake in gastric, duodenal, and colorectal cancers. Radiology 298:393–402 33258746 10.1148/radiol.2020203275

[93] Furtado FS, Wu MZ, Esfahani SA et al (2023) Positron emission tomography/magnetic resonance imaging (PET/MRI) versus the standard of care imaging in the diagnosis of peritoneal carcinomatosis. Ann Surg 277:e893–e899 35185121 10.1097/SLA.0000000000005418 PMC11346589

[94] Dresen RC, De Vuysere S, De Keyzer F et al (2019) Whole-body diffusion-weighted MRI for operability assessment in patients with colorectal cancer and peritoneal metastases. Cancer Imaging 19:1 30616608 10.1186/s40644-018-0187-z PMC6322317

[95] Rajendran K, Petersilka M, Henning A et al (2022) First clinical photon-counting detector CT system: technical evaluation. Radiology 303:130–138 34904876 10.1148/radiol.212579 PMC8940675

[96] Kim TM, Kim SY, Cho JY, Kim SH, Moon MH (2020) Utilization of virtual low-keV monoenergetic images generated using dual-layer spectral detector computed tomography for the assessment of peritoneal seeding from ovarian cancer. Medicine (Baltimore) 99:e20444 32501991 10.1097/MD.0000000000020444 PMC7306341

[97] Thivolet A, Si-Mohamed S, Bonnot PE et al (2020) Spectral photon-counting CT imaging of colorectal peritoneal metastases: initial experience in rats. Sci Rep 10:13394 32770125 10.1038/s41598-020-70282-w PMC7414131

