# Supplementary material for: CT and MR in peritoneal malignancies: pearls and pitfalls at preoperative examination
Source: Insights Imaging. 2025 Aug 8;16:171. doi: 10.1186/s13244-025-02060-z (PMC12334402; doi:10.1186/s13244-025-02060-z)

Bone reporting and data system on CT (Bone-RADS-CT): a validation study by four readers on 328 cases from three local and two public databases

ELECTRONIC SUPPLEMENTARY MATERIAL

Figure S1:

Graphical representation of peritoneal cancer index score.

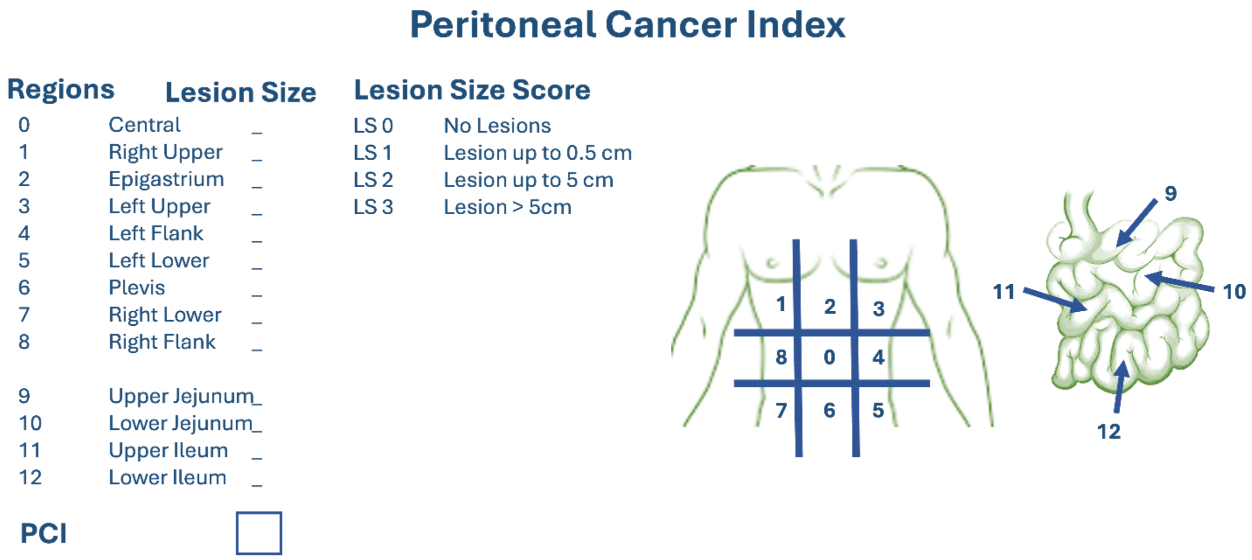

Figure S2:

Tuberculosis reactivation in 72-year-old man affected by lung cancer treated with immunotherapy. CT portal venous phase, axial plane **(A-B)** showed a regular thickening of the peritoneum **(A-B, arrowhead)**, in association with ileal thickening **(A, asterisks)** and ileo-ileal fistula **(B circle)**. Coronal plane **(C)** confirmed the linear thickening of the peritoneum **(C, arrowhead),** and ileo-ileal fistula (**B circle).** Necrotic nodes were also present in left iliac fossa **(A and C, arrows).**

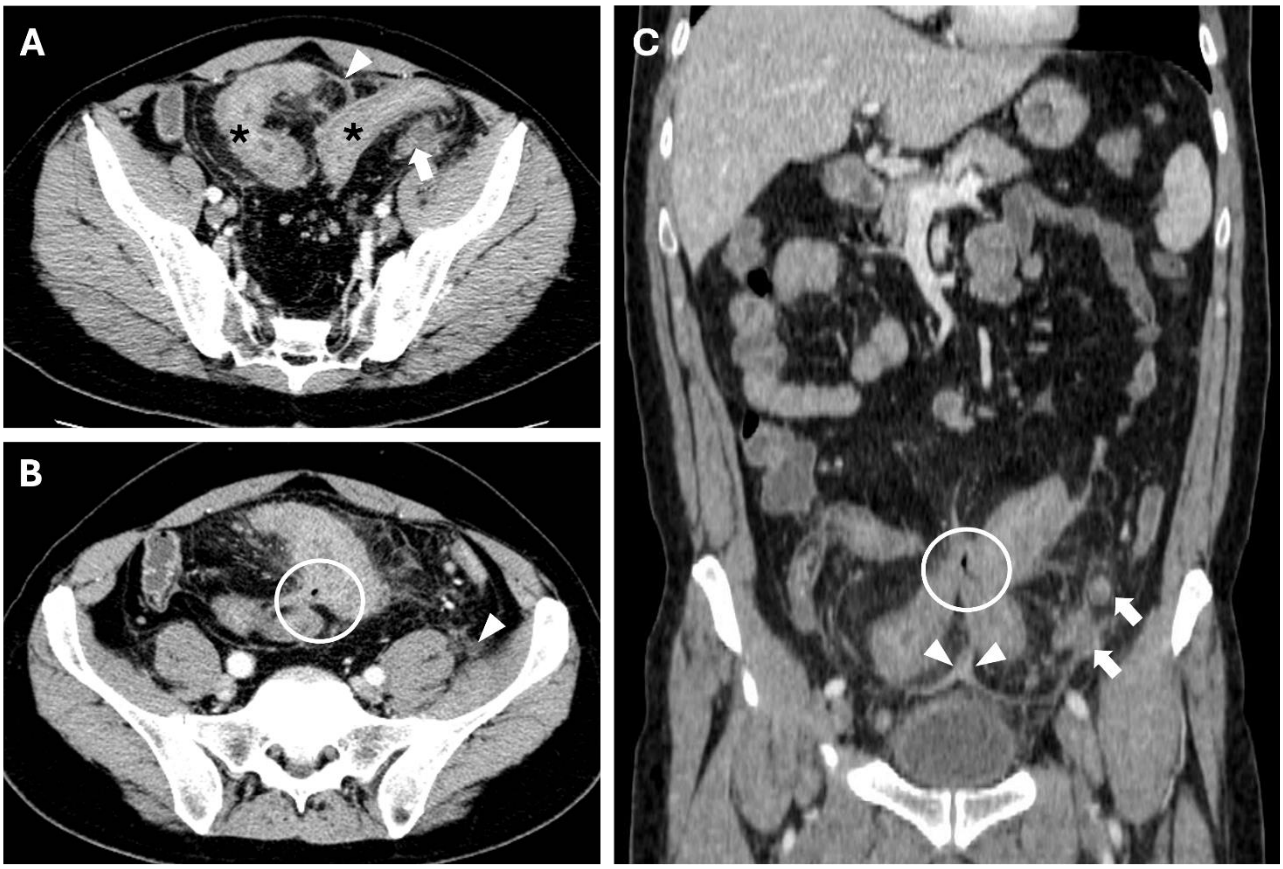

Figure S3:

Abdominal splenosis in 57-year-old man affected by lung cancer treated with surgery. CT late arterial **(A-C)** and portal venous phases **(D-F)** on axial plane showed nodular implants following the typical spleen enhancement **(A-F, arrows)**.

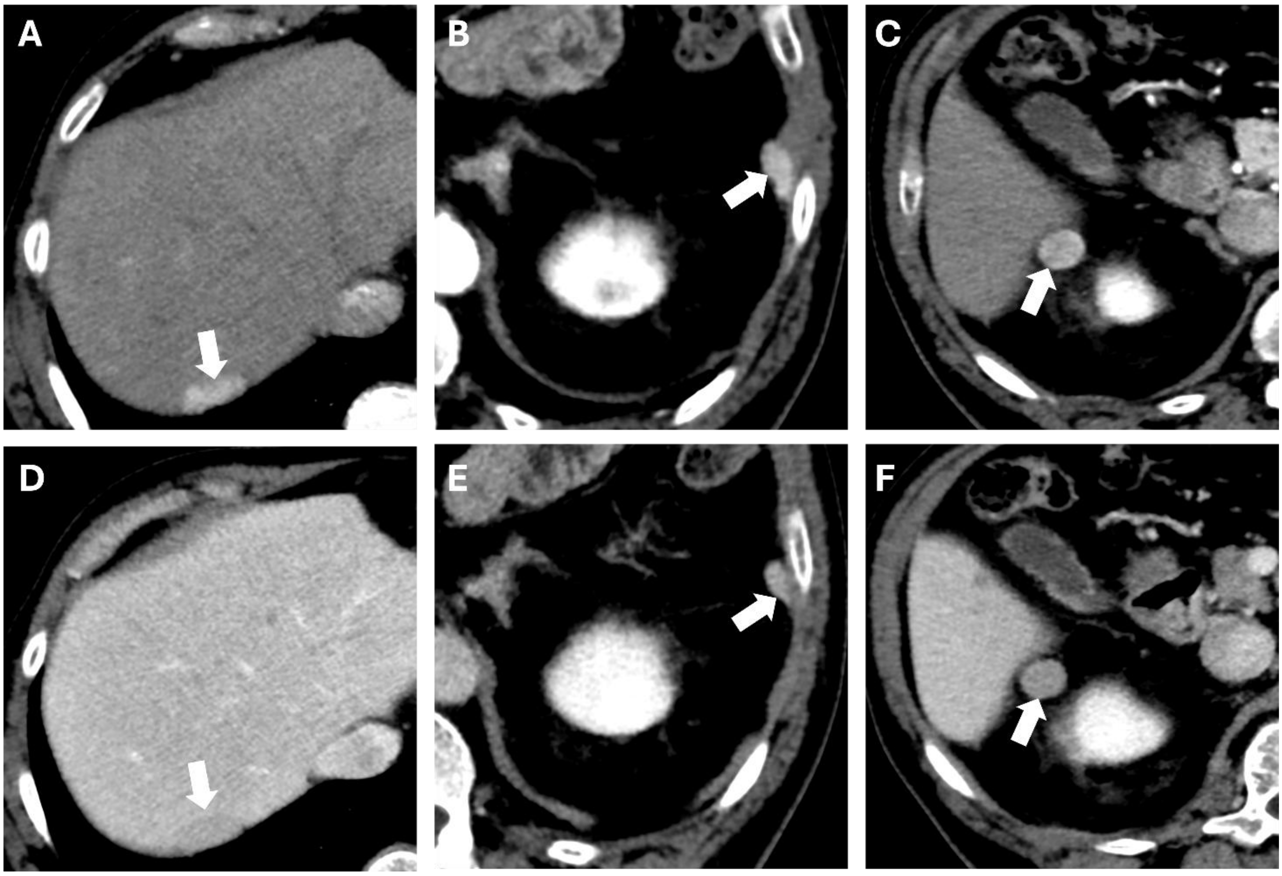

Figure S4:

Ileal lymphoma in 48-year-old woman with peritoneal lymphomatosis. CT portal venous phase, axial plane **(A-C)** showed a linear **(A, arrowheads)** and nodular **(A and C, circle)** thickening of the peritoneum, in association with ileal thickening **(B, arrows)** with pseudo-aneurysmatic pattern and necrotic adenopathy **(A, asterisk)**.

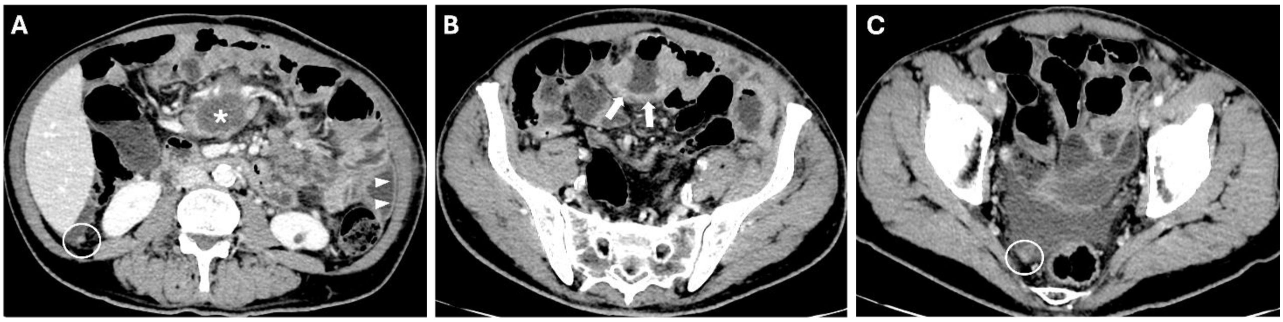

Figure S5:

Malignant mesothelioma in 55-year-old woman. CT portal venous phase **(A-D)**, axial plane **(A and E)** showed cystic **(A, asterisk)** and nodular implants **(E, arrow)**. Cystic implant was hyperintense in MRI T2w **(D, asterisk)** without restricted diffusion on DWI (b=800 mm2/s) and ADC map **(B and C, asterisks)**. Nodular implant was hypointense in MRI T2w **(H, asterisk)** with restricted diffusion on DWI (b=800 mm2/s) and ADC map **(F and G, asterisks)**.

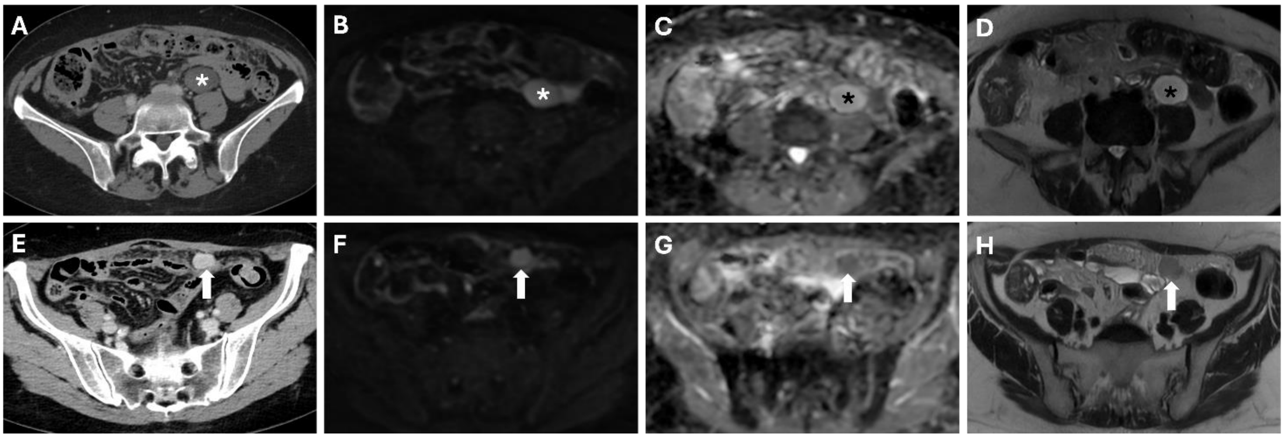

Figure S6:

Endometriosis in 42-year-old woman with history of appendiceal tumor. MRI T2w **(A)** and T1fs **(B)** showed ovarian endometriomas with recent **(A and B, asterisks)** and remote **(A and B, arrowheads)** signs of bleeding in association with nodule of adenomyosis **(A, circle)**.

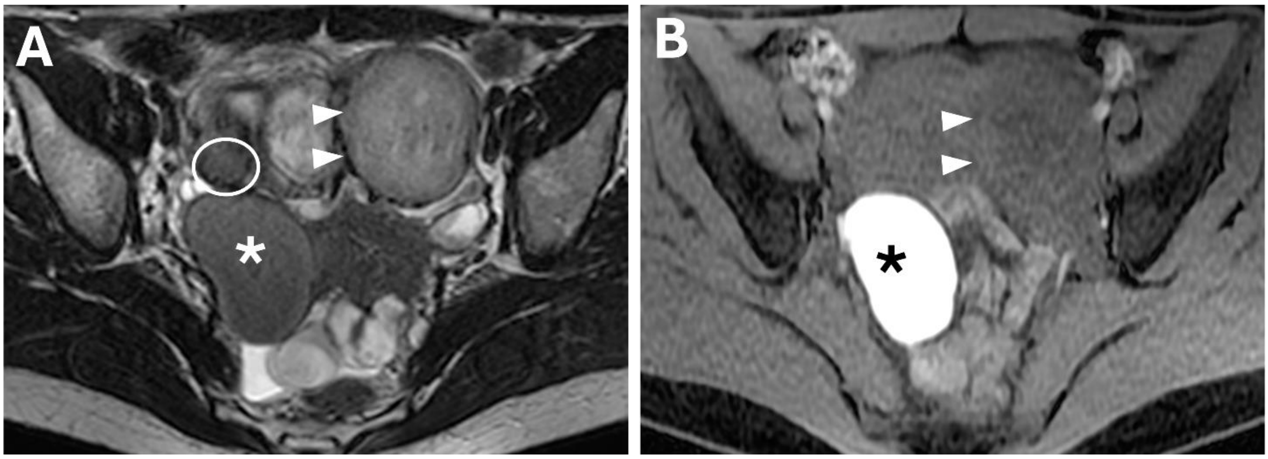

Figure S7:

Graphical representation of the four main surgical needs.

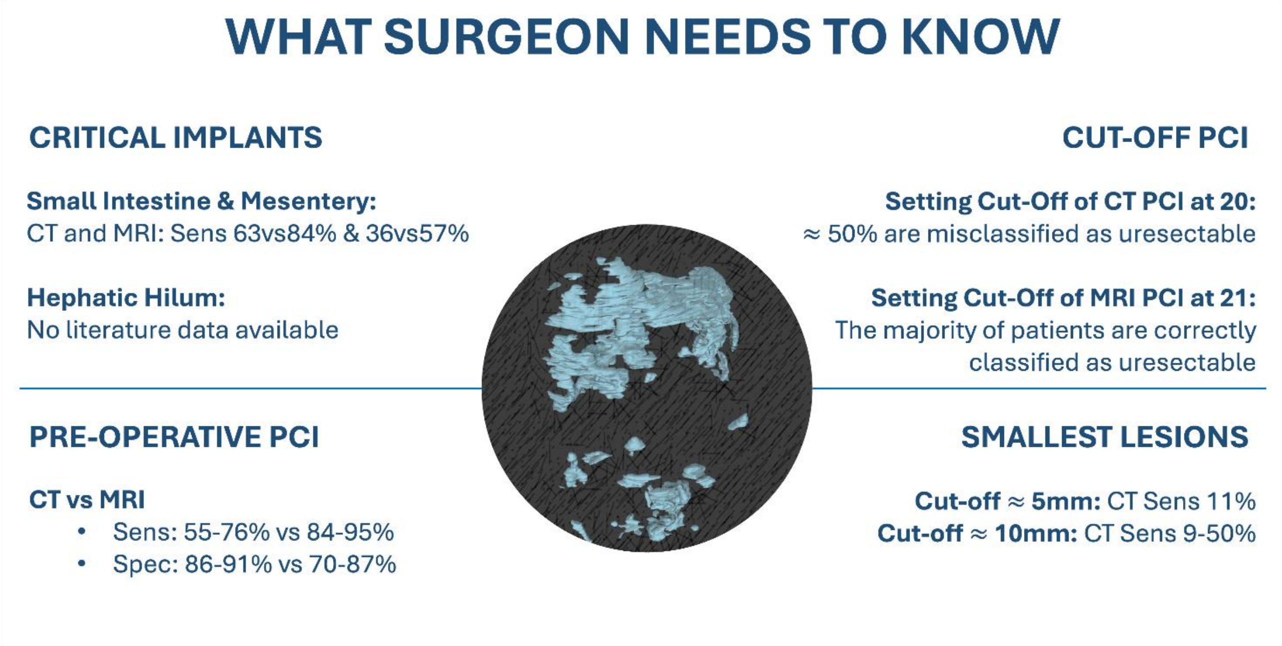

Supplement: Supplementary file 1 — ELECTRONIC SUPPLEMENTARY MATERIAL [file 13244_2025_2060_MOESM1_ESM.pdf]
